# Supplementary material for: The radiation protection behavior of medical workers: A scoping review protocol
Source: PLoS One. 2024 Aug 6;19(8):e0308479. doi: 10.1371/journal.pone.0308479 (PMC11302849; doi:10.1371/journal.pone.0308479)
Supplement: S1 Checklist — (DOCX) [file pone.0308479.s001.docx]

**Preferred Reporting Items for Systematic reviews and Meta-Analyses extension for Scoping Reviews (PRISMA-ScR) Checklist**

| SECTION | ITEM | PRISMA-ScR CHECKLIST ITEM | REPORTED ON PAGE |
| --- | --- | --- | --- |
| Title | 1 | Identify the report as a scoping review. | P1 |
| Executive  summary | 2 | Provide a structured summary that includes (as applicable): background, objectives, eligibility criteria, sources of evidence, charting methods, results, and conclusions that relate to the review questions and objectives. | P1 |
| Background | 3 | Describe the rationale for the review in the context of what is already known. | P1-2 |
| Objectives | 4 | Provide an explicit statement of the questions and objectives being addressed with reference to their key elements (e.g., population or participants, concepts, and context) or other relevant key elements used to conceptualize the review questions and/or objectives. | P2 |
| Protocol and registration | 5 | Indicate whether a review protocol exists; state if and where it can be accessed (e.g., a Web address); and if available, provide registration information, including the registration number. | P3 |
| Eligibility criteria | 6 | Specify characteristics of the sources of evidence used as eligibility criteria (e.g., years considered, language, and publication status), and provide a rationale. | P3-4 |
| Information sources | 7 | Describe all information sources in the search (e.g., databases with dates of coverage and contact with authors to identify additional sources), as well as the date the most recent search was executed. | P3 |
| Search strategy | 8 | Present full electronic search strategy for at least one database, including any limits used, such that it could be repeated. | P3 |
| Study selection process | 9 | State the process for selecting sources of evidence (i.e., screening and eligibility) included in the scoping review. | P3 |
| Data collection process | 10 | Describe the methods of charting data from the included sources of evidence (e.g., calibrated forms or forms that have been tested by the team before their use, and whether data charting was done independently or in duplicate) and any processes for obtaining and confirming data from investigators. | P5 |
| Data items | 11 | List and define all variables for which data were sought and any assumptions and simplifications made. | P5 |
| Risk of bias assessment of individual studies | 12 | Describe methods used for assessing risk of bias of individual studies (including specification of whether this was done at the study or outcome level), and how this information is to be used in any data synthesis. | / |
| Summary measures | 13 | State the principal summary measures (e.g., risk ratio, difference in means). | / |
| Data synthesis methods | 14 | Describe the methods of handling data and combining results of studies. | / |
| Risk of bias assessment across studies | 15 | Specify any assessment of risk of bias that may affect the cumulative evidence. | / |
| Additional analyses | 16 | Describe methods of additional analyses (e.g., sensitivity or subgroup analyses, meta regression), if done, indicating which were pre-specified. | / |
| Study selection results | 17 | Give numbers of studies screened, assessed for eligibility, and included in the review, with reasons for exclusions at each stage, ideally with a flow diagram. | / |
| Study characteristics | 18 | For each study, present characteristics for which data were extracted. | / |
| Results of risk of bias within studies | 19 | Present data on risk of bias of each study and, if available, any outcome level assessment (see item 12). | / |
| Results of individual studies | 20 | For all outcomes considered (benefits or harms), present, for each study: (1) simple summary data for each intervention group; (2) effect estimates and confidence intervals, ideally with a forest plot | / |
| Data synthesis results | 21 | Summarize and/or present the charting results as they relate to the review questions and objectives. | / |
| Results of risk of bias across studies | 22 | Present results of any assessment of risk of bias across studies (see item 15). | / |
| Results from additional analyses | 23 | Give results of additional analyses, if done (e.g., sensitivity or subgroup analyses, meta-regression [see item 16]). | / |
| Discussion (summary of evidence) | 24 | Summarize the main findings including the strength of evidence for each main outcome; consider their relevance to key groups (e.g., health care providers, users, and policy makers). | / |
| Limitations | 25 | Discuss the limitations of the scoping review process. | P6 |
| Conclusions | 26 | Provide a general interpretation of the results in the context of other evidence, and implications for future research. | / |
| Funding source(s) | 27 | Describe sources of funding for the included sources of evidence, as well as sources of funding for the scoping review. Describe the role of the funders of the scoping review. | P6 |
