# Supplementary material for: The radiation protection behavior of medical workers: A scoping review protocol
Source: PLoS One. 2024 Aug 6;19(8):e0308479. doi: 10.1371/journal.pone.0308479 (PMC11302849; doi:10.1371/journal.pone.0308479)
Supplement: S1 Table — (DOCX) [file pone.0308479.s003.docx]

| # | Searches | Results |
| --- | --- | --- |
| 1 | (("Medical Staff"[Mesh]) OR "Health Personnel"[Mesh]) OR "Medical Staff, Hospital"[Mesh] | 631,301 |
| 2 | (((((((((((hospitalist) OR (hospitalists)) OR (nurse)) OR (nurses)) OR (doctors)) OR (doctor)) OR (radiologist)) OR (radiologists)) OR (dentist)) OR (dentists)) OR (surgeon)) OR (surgeons) | 1,536,687 |
| 3 | 1 OR 2 | 1,784,726 |
| 4 | ((((behavior) OR (behaviors)) OR (practice)) OR (practices)) OR (compliance) | 6,460,105 |
| 5 | ((radiation protection) OR (radiation safety)) OR (radiation protective) | 148,290 |
| 6 | 4 AND 5 | 21,013 |
| 7 | 3 AND 6 | 1,499 |
| Filters: Full text, from 2010/1/1-2023/12/31 | | |

**Search strategy for PUBMED**
