# Supplementary material for: The radiation protection behavior of medical workers: A scoping review protocol
Source: PLoS One. 2024 Aug 6;19(8):e0308479. doi: 10.1371/journal.pone.0308479 (PMC11302849; doi:10.1371/journal.pone.0308479)
Supplement: Attachment — Submitted filename: Review.docx [file pone.0308479.s004.docx]

The study entitled “The radiation protection behavior of medical workers: A scoping review protocol” This study collects information about Definition of radiation protection behavior, including aspects such as current practices of radiation protection in medical settings and the instruments used for measurement. The study aims to examine the current research status of radiation protection behaviours. To identify research gaps and guide future research directions.

**This manuscript well organised and it useful for medical field and their workers, However, some suggestions can be considered for minor revision to get published in the journal.**

1) **Kindly check the Kindly check the grammar and English throughout the manuscript.**
2) **Add some points in the discussion part**
3) **Add some recent reference**
