# Supplementary material for: The radiation protection behavior of medical workers: A scoping review protocol
Source: PLoS One. 2024 Aug 6;19(8):e0308479. doi: 10.1371/journal.pone.0308479 (PMC11302849; doi:10.1371/journal.pone.0308479)
Supplement: Attachment — Submitted filename: Response to Reviewers.docx [file pone.0308479.s005.docx]

Dear reviewer:

Thank you for your decision and constructive comments on my manuscript. We have carefully considered the suggestions of the Reviewers and made some changes. We have tried our best to improve and made some changes to the manuscript. The yellow part has been revised according to your comments. Revision notes, point-to-point, are given as follows:

Reviewer#1:

1) Kindly check the Kindly check the grammar and English throughout the manuscript.

We were really sorry for our careless mistakes. Thanks for your reminder. We have tried our best to revise the mistake of grammar and English in the revised manuscript.

2) Add some points in the discussion part

As suggested by the reviewer, we have added more points in the discussion part. (P5 Line32-40, P6 Line1-26 marked in yellow in the revised paper)

3) Add some recent reference

We have replaced some old references and added some recent references. (P9-P10 marked in yellow in the revised paper)

Reviewer#2:

1) The authors have not included the number of patients or participants (n =?) in Figure 1. Please explain

We are sorry for this careless mistake, and we have completed the number in Figure 1. in the revised paper.

2) The authors kindly include the study's conclusion.

As suggested by the reviewer, we have added the study's conclusion. (P6 Line27-34 marked in yellow in the revised paper)

3) In the discussion section, the authors should discuss this study more in relation to previous studies (please add references).

We have discussed this study more in relation to previous studies and added more references. (P5 Line32-40, P6 Line1-26 marked in yellow in the revised paper)

Thanks again for your professional review work on our article. If you have any queries, please don’t hesitate to contact me at the address below.

Yours sincerely,

Xun Liu

School of Nursing, University of South China, Hengyang, Hunan Province, China

LX13097380215@outlook.com
